# Acan downregulation in parvalbumin GABAergic cells reduces spontaneous recovery of fear memories

**DOI:** 10.1038/s41380-023-02085-0

**Published:** 2023-05-02

**Authors:** Marisol Lavertu-Jolin, Bidisha Chattopadhyaya, Pegah Chehrazi, Denise Carrier, Florian Wünnemann, Séverine Leclerc, Félix Dumouchel, Derek Robertson, Hicham Affia, Kamal Saba, Vijaya Gopal, Anant Bahadur Patel, Gregor Andelfinger, Graçiela Pineyro, Graziella Di Cristo

**Affiliations:** 1 Centre de Recherche, CHU Sainte-Justine (CHUSJ), Montréal, QC Canada; 2 Department of Neurosciences, Université de Montréal, Montréal, QC Canada; 3 CSIR-Centre for Cellular and Molecular Biology, Hyderabad, 500007 India; 4 Academy of Scientific and Innovative Research (AcSIR), Ghaziabad, 201002 India; 5 Department of Pediatrics, Université de Montréal, Montréal, QC Canada; 6 Department of Pharmacology, Université de Montréal, Montréal, QC Canada; 7 Present Address: Heidelberg University, Faculty of Medicine & Heidelberg University Hospital, Institute for Computational Biomedicine, Bioquant, Heidelberg, Germany

**Keywords:** Neuroscience, Drug discovery, Psychiatric disorders

## Abstract

While persistence of fear memories is essential for survival, a failure to inhibit fear in response to harmless stimuli is a feature of anxiety disorders. Extinction training only temporarily suppresses fear memory recovery in adults, but it is highly effective in juvenile rodents. Maturation of GABAergic circuits, in particular of parvalbumin-positive (PV^+^) cells, restricts plasticity in the adult brain, thus reducing PV^+^ cell maturation could promote the suppression of fear memories following extinction training in adults. Epigenetic modifications such as histone acetylation control gene accessibility for transcription and help couple synaptic activity to changes in gene expression. Histone deacetylase 2 (Hdac2), in particular, restrains both structural and functional synaptic plasticity. However, whether and how Hdac2 controls the maturation of postnatal PV^+^ cells is not well understood. Here, we show that PV^+^- cell specific *Hdac2* deletion limits spontaneous fear memory recovery in adult mice, while enhancing PV^+^ cell bouton remodeling and reducing perineuronal net aggregation around PV^+^ cells in prefrontal cortex and basolateral amygdala. Prefrontal cortex PV^+^ cells lacking *Hdac2*, show reduced expression of *Acan*, a critical perineuronal net component, which is rescued by Hdac2 re-expression. Pharmacological inhibition of Hdac2 before extinction training is sufficient to reduce both spontaneous fear memory recovery and *Acan* expression in wild-type adult mice, while these effects are occluded in PV^+^-cell specific *Hdac2* conditional knockout mice. Finally, a brief knock-down of *Acan* expression mediated by intravenous siRNA delivery before extinction training but after fear memory acquisition is sufficient to reduce spontaneous fear recovery in wild-type mice. Altogether, these data suggest that controlled manipulation of PV^+^ cells by targeting Hdac2 activity, or the expression of its downstream effector *Acan*, promotes the long-term efficacy of extinction training in adults.

## Introduction

Anxiety and trauma-related disorders are associated with a huge socioeconomic burden, given the limited treatment options available [1]. Certain anxiety disorders are characterized by abnormally persistent emotional memories of fear-related stimuli, and impaired inhibition of learned fear is a feature in post-traumatic stress disorder (PTSD), anxiety disorders and phobias [2]. In rodents, pairing a neutral tone (conditioned stimulus, CS) with an aversive foot shock (unconditioned stimulus, US) leads to the formation of a robust fear memory that can last an entire lifetime [3]. The reduction of fear responses through fear extinction learning is at the heart of clinical exposure psychotherapies applied in the case of PTSD [4, 5]. In adults, extinction learning, or the gradual decrease of behavioral response to a CS that occurs when the stimulus is presented without reinforcement (e.g., without the aversive foot shock), is neither robust nor permanent since conditioned fear responses can recover spontaneously with the passage of time; in other words, extinction learning is unstable in adults [3, 6, 7]. Strategies aimed at enhancing the ability to inhibit responses to associations that are no longer relevant may have strong therapeutic value. Several studies suggested that the maturation of GABAergic neurotransmission contributes to the increased spontaneous recovery of fear memory following extinction learning in adults [8, 9]. Therefore, modulating GABAergic function is an attractive target to reduce spontaneous recovery of fear memories with time after extinction training in adults. However, global or prolonged reduction of GABAergic drive can generate undesirable effects such as epileptic activity. Therefore, ideal approaches targeting GABAergic transmission to foster adult brain plasticity need to be both cell type-specific and temporally controlled.

Amongst the different GABAergic interneuron types, reducing parvalbumin-expressing (PV^+^) interneuron activity or connectivity is sufficient to reinstate heightened plasticity in adult sensory cortices [10–13]. Recent studies showed that PV^+^ cells also play a role in fear memory extinction. First, fear responses during extinction learning are increased by chemogenetic activation of PV^+^ cells in the medial prefrontal cortex [14]. Second, extinction training induces remodeling of perisomatic PV^+^ synapses around excitatory neurons that were previously activated during fear conditioning in the basolateral amygdala (BLA) [15]. Third, PV^+^ cell-specific deletion of Nogo Receptor 1, a neuronal receptor for myelin-associated growth inhibitors [16], enhances BLA PV^+^ synapse remodeling and reduces the spontaneous recovery of fear memory after extinction training [17]. Fourth, fear extinction weakens the functional outputs of PV^+^ cells to pyramidal neurons in medial prefrontal cortex [14]. All together, these data suggest that GABAergic PV^+^ synapse remodeling, an activity-regulated process [18–20], may be implicated in extinction learning and long-term spontaneous recovery of fear memories.

Transcriptional mechanisms coupling synaptic activity to changes in gene expression drive cellular processes that mediate behavioral adaptations [21]. Accessibility of the chromatin to activate these transcriptional programs is modulated by changes in the state of histone acetylation of chromatin. In particular, blocking endogenous histone deacetylases (Hdacs) promotes both structural and functional synaptic plasticity processes [22, 23] that are required to modify behavior and improve performance during learning. Accordingly, administration of pan-Hdac inhibitors in adult mice reactivates visual cortical plasticity [24] and enhances extinction learning [25]. Several studies have shown that, of the many Hdacs expressed in the mammalian brain, Hdac2 plays a critical role in fear learning and memory [22, 23, 26]. A recent study showed that PV^+^ cell-specific deletion of Hdac2 was sufficient to decrease evoked IPSC and increase long-term depression in layer 2-3 of adult visual cortex [27], suggesting that deletion of Hdac2 in PV^+^ cells induced juvenile-like phenotypes both in inhibition and cortical plasticity. Therefore, manipulating Hdac2 expression in PV^+^ cells is an effective strategy to reduce their inhibitory output. Whether this would be sufficient to attenuate the recovery of fear memories after extinction training in adults is unknown.

PV^+^ interneurons are the only cortical cell type surrounded by perineuronal nets (PNNs), which are lattice-like aggregates of chondroitin sulfate proteoglycans-containing extracellular matrix [20]. The percentage of PV^+^ interneurons enwrapped by PNN increases during development in different brain regions, including those involved in fear memory formation and extinction [28]. Recent evidences show that PNNs stabilize afferent synaptic contacts onto mature cortical PV^+^ cells, thus limiting their plasticity [20, 29, 30]. Indeed, degradation of PNNs in adult BLA promotes fear memory erasure [28], a phenomena observed only in younger animals [8, 28, 31]. PNNs are dynamic structures, comprising of numerous structural components and remodeled by metalloproteases [32]. Whether the expression of distinct PNN components is controlled by epigenetic regulation specifically in PV^+^ cells, in turn modulating their role in cortical plasticity, is unknown.

Here, we report that *Hdac2* deletion or pharmacological inhibition in PV^+^ cells attenuate spontaneous recovery of fear memory after fear extinction learning in adults. In particular, these manipulations promote a temporally downregulation of *Acan*, coding for aggrecan, a critical perineuronal net component, which we show to be expressed almost exclusively by PV^+^ cells in medial prefrontal cortex. Finally, we show that intravenous delivery of siRNA against *Acan* right before extinction training induces knock-down of *Acan* expression in prefrontal cortical PV^+^ cells and reduces spontaneous fear recovery with time in adult wild-type mice. Therefore, transiently changing the expression of a critical PNN component specifically in PV^+^ cells before extinction training but after fear memory acquisition is sufficient to reduce spontaneous fear memory recovery in adult mice.

## Results

### Hdac2 inhibition, in PV^+^ cells, leads to reduced spontaneous recovery of fear memory with time following extinction training in adult mice

**Fig. 1:**
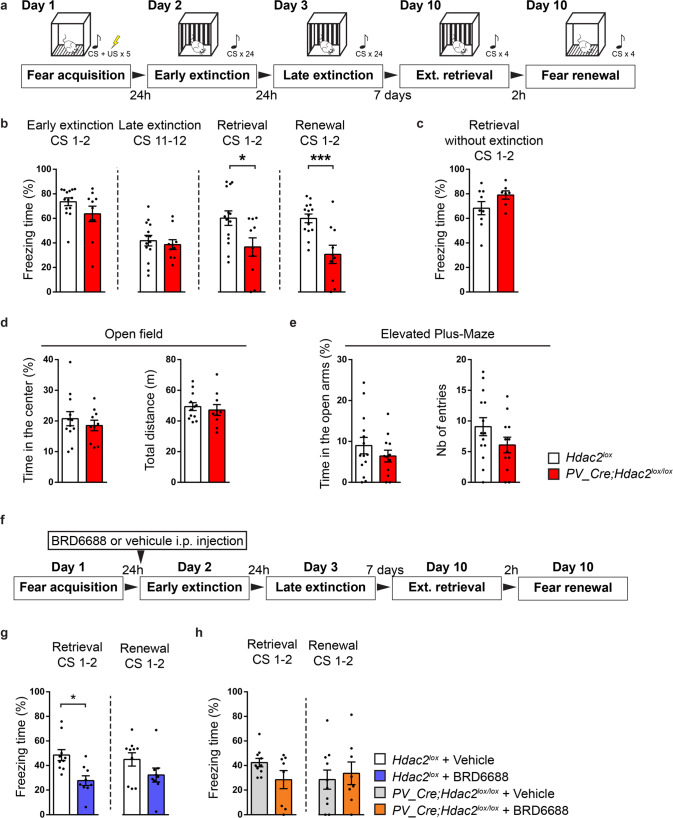
Postnatal *Hdac2* deletion restricted to PV^+^ cells or Hdac2 inhibition before extinction training decrease the spontaneous recovery of fear memories after extinction in adult mice. **a** Schematic representation of the experimental protocol. Ext. extinction. **b** Wild-type and *PV-Cre; Hdac2*^*lox/lox*^ littermates show efficient fear extinction. Repeated two-way ANOVA; F_genotype_ (1,22) = 4.430, *P* = 0.0470, F_extinction_(11,242) = 8.864, *P* < 0.0001, F_genotype_*_extinction_ (11,242) = 0.6427, *P* = 0.7911. Wild-type, but not *PV-Cre; Hdac2*^*lox/lox*^ littermates, show spontaneous recovery and context-dependent renewal of fear memory. Unpaired two-tailed *t*-tests, *P* = 0.0205 for fear retrieval; *P* = 0.0008 for fear renewal. Number of mice: *Hdac2*^*lox/+*^ or *Hdac2*^*lox/lox*^ *n* = 14; *PV-Cre; Hdac2*^*lox/lox*^ *n* = 10. **c** In the absence of extinction training, *PV-Cre; Hdac2*^*lox/lox*^ mice show stable fear memory 10 days after conditioning, as control littermates (Mann–Whitney test*, P* = 0.2509). Number of mice: *Hdac2*^*lox/+*^ or *Hdac2*^*lox/lox*^ *n* = 9; *PV-Cre; Hdac2*^*lox/lox*^ *n* = 7. **d** In the open field test, there is no significant difference in time spent in the center of the open field (unpaired two-tailed *t*-test, *P* = 0.4635) nor the total distance traveled (unpaired two-tailed *t*-test*, P* = 0.6083) between the two genotypes. Number of mice: *Hdac2*^*lox/+*^ or *Hdac2*^*lox/lox*^ *n* = 12; *PV-Cre; Hdac2*^*lox/lox*^ *n* = 10. **e** In the elevated plus maze test, there is no genotype-dependent difference in time spent (unpaired two-tailed *t*-test, *P* = 0.3279) nor the number of entries in the open arms (unpaired two-tailed *t*-test, *P* = 0.1449). Number of mice: *Hdac2*^*lox/+*^ or *Hdac2*^*lox/lox*^ *n* = 14; *PV-Cre; Hdac2*^*lox/lox*^ *n* = 12. **f** Schematic representation of the experimental protocol. Hdac2 inhibitor BRD6688 or the vehicle was injected intraperitoneally (i.p.) 6 h before the early extinction procedure. **g** One week after extinction training, freezing levels are significantly different in the spontaneous recovery test between vehicle- and BRD6688-injected *Hdac2*^*lox/lox*^ (**h**) but not between vehicle- and BRD6688-injected *PV-Cre; Hdac2*^*lox/lox*^ mice. **g** Extinction retrieval unpaired two-tailed *t*-test, *P* = 0.0030; fear renewal, unpaired two-tailed *t*-test, *P* = 0.1246. Number of *Hdac2*^*lox/lox*^ mice injected with vehicle, *n* = 10 or BRD6688, *n* = 9. **h** Extinction retrieval unpaired two-tailed *t*-test *P* = 0.0850, fear renewal unpaired two-tailed *t*-test *P* = 0.6733. Number of *PV-Cre; Hdac2*^*lox/lox*^ mice injected with vehicle, *n* = 10 or BRD6688, *n* = 8. Graph bars represent mean ± s.e.m. Circles represent individual mouse values. CS Conditioned stimulus, US Unconditioned stimulus. *P < 0.05, ***P < 0.001.

To evaluate whether and how *Hdac2* deletion specifically in PV^+^ cells affects fear memory formation, extinction and spontaneous recovery, we crossed mice carrying a conditional allele of *Hdac2* (*Hdac2*^*lox/lox*^, [22]), which allows cell-specific developmental stage-restricted manipulation of *Hdac2*, with mice expressing Cre recombinase under the control of the PV promoter (*PV-Cre*, [33]). In cortical GABAergic cells, PV starts to be expressed after the first postnatal week and peaks after the third week. This breeding scheme generated PV^+^-cell restricted homozygous (*PV-Cre*^*+/−*^;*Hdac2*^*lox/lox*^) mice and control *PV-Cre*^*−/−*^ littermates (*PV-Cre*^*−/−*^;*Hdac2*^*lox/lox*^ and *PV-Cre*^*−/−*^;*Hdac2*^*lox/+*^ mice, referred to hereafter as *Hdac2*^*lox*^). To confirm the specificity of Cre expression in *PV-Cre*^*+/−*^*;Hdac2*^*lox/lox*^ mice, we bred them with *RCE*^*GFP*^ reporter mice [34] and quantified GFP^+^/PV^+^ colocalized cells in somatosensory cortex (SSCX), medial prefrontal cortex (PFC) and basolateral amygdala (BLA). At P60, we observed that all GFP^+^ cells in all brain regions studied expressed PV, whereas the recombination rate was region specific (GFP^+^PV^+^/PV^+^ cells: SSCX: 82 ± 5%, *n* = 3 mice; PFC: 58 ± 8%, *n* = 5 mice; BLA: 38 ± 3%, *n* = 3 mice). Co-immunolabelling of PV and Hdac2 in coronal brain sections confirmed that Hdac2 expression levels were significantly reduced in PV^+^ cells in the conditional KO mice compared to their control littermates (Supplementary Fig. 1). We then assessed fear memory formation, extinction rate, spontaneous recovery and fear renewal in *PV-Cre*; *Hdac2*^*lox/lox*^ mice (Fig. 1a). After fear conditioning, both *PV-Cre*; *Hdac2*^*lox/lox*^ and *Hdac2*^*lox*^ mice presented strong freezing behavior, suggesting that fear learning was not affected by *Hdac2* deletion in PV^+^ cells (freezing time at early extinction (CS1-2), Mann–Whitney test, *P* = 0.1878, Fig. 1b). Compared to wild-type littermates, conditional KO mice showed similar extinction rate during training (Fig.1b). One week after extinction training, mice were re-exposed to the conditional stimulus (CS) either in the extinction context (retrieval test, to evaluate spontaneous fear memory recovery) or in the fear acquisition context (renewal test, to evaluate for generalization of extinction learning). Compared to wild-type littermates, conditional KO mice showed significantly less freezing both during the retrieval and renewal tests 7 days later (Fig. 1b), suggesting reduced spontaneous recovery of fear memory. The reduced fear expression was not due to loss of memory, since in absence of extinction training, freezing levels were undistinguishable between conditional KO and control mice 10 days after fear conditioning (Fig. 1c). Further, this was not due to altered motor or anxiety behavior, since we found no differences between the genotypes in the open field and elevated plus maze assays (Fig. 1d, e).

To ensure that the observed phenotype was caused by the genetic deletion of *Hdac2* rather than any side-effect of the *Cre* insertion in the *PV* locus [33], we generated a second mouse line in which control littermates also carried the *PV-Cre* allele, while the *Hdac2* locus remained wild-type (*PV-Cre*^*+/−*^*;Hdac2*^*+/+*^ mice). Consistent with our previous observations, these conditional KO mice showed significantly reduced freezing during the retrieval, but not the renewal, test compared to their control littermates (Supplementary Fig. 2a). In absence of extinction training, both genotypes showed strong fear memory 10 days after fear learning (Supplementary Fig. 2b). Overall, these data suggest that the deletion of *Hdac2* specifically in postnatal PV^+^ cells is sufficient to reduce spontaneous recovery of fear memories over time in adults.

Reduced spontaneous recovery of fear memories after extinction training in adult *PV-Cre*; *Hdac2*^*lox/lox*^ mice could rely on different mechanisms recruited during fear memory acquisition in the conditional KO mice compared to control littermates. For example, Gogolla et al. [28] showed that enzymatic degradation of PNN in the BLA of adult mice before but not after fear memory acquisition increased fear memory erasure susceptibility. Alternatively, *Hdac2* deletion in PV^+^ cells may specifically affect the long-term retention of extinction memories. To distinguish between these two hypotheses, we used BRD6688, a small molecule with kinetic selectivity for Hdac2 as compared to Hdac1, a close family member showing 92% homology within the catalytic domain with Hdac2 [35, 36]. We first asked whether pharmacological inhibition of Hdac2 after fear learning but before the onset of extinction training was sufficient to reduce spontaneous fear memory recovery in wild-type mice (Fig. 1f). Compared to vehicle-injected mice, BRD6688-injected mice presented less freezing behavior at the retrieval, but not at the renewal, test (Fig. 1g). Therefore, combining a brief Hdac2 inhibition with extinction training rendered the extinction memory stronger, increasing its persistence over time. Circuits other than PV^+^ cells may contribute to the promoting effect of Hdac2 inhibition on the reduced spontaneous recovery of fear memory after extinction training. If the observed effect was mostly mediated by Hdac2 expression in PV^+^ cells, we would expect that spontaneous recovery of fear memory in wild-type mice would be reduced by Hdac2 inhibition, whereas the same treatment should have no effects in conditional KO mice. We found this to be the case; indeed, *PV-Cre; Hdac2*^*lox/lox*^ mice injected with either vehicle or BRD6688 did not show any significant difference in the freezing behavior during the retrieval test (Fig. 1h), thus supporting the hypothesis that the state of chromatin acetylation regulated by Hdac2 in PV^+^ cells plays a critical role in limiting the spontaneous recovery of fear memory with time after extinction training.

### Postnatal deletion of *Hdac2* in PV^+^ cells enhances the remodeling of their synaptic terminals

**Fig. 2:**
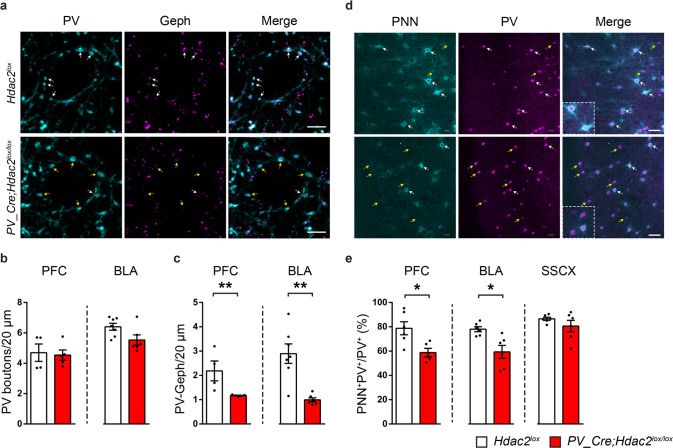
*PV_Cre; Hdac2*^*lox/lox*^ mice show decreased PV^+^ cell efferent synaptic connectivity and reduced PNN agglomeration around cortical PV^+^ cell bodies. **a** PFC coronal sections of *PV-Cre; Hdac2*^*lox/lox*^ and control littermates immunolabeled for PV (cyan) and gephyrin (magenta). White arrows indicate perisomatic PV-positive boutons colocalizing with Gephyrin (PV^+^Gephyrin^+^ boutons), while yellow arrows indicate perisomatic PV-positive boutons that do not colocalise with Gephyrin (PV^+^Gephyrin^-^ boutons). Scale bar, 5 µm. **b** The density of perisomatic PV^+^ boutons is not significantly different neither in PFC nor in BLA between the two genotypes. PFC: Mann–Whitney test, *P* = 0.6825, *Hdac2*^*lox/lox*^ *n* = 4; *PV-Cre; Hdac2*^*lox/lox*^ *n* = 5;. BLA: Mann–Whitney test, *P* = 0.1014, *Hdac2*^*lox/lox*^ *n* = 7; *PV-Cre; Hdac2*^*lox/lox*^ *n* = 6. **c** The density of perisomatic PV^+^ boutons co-localizing with gephyrin puncta is significantly reduced in both PFC and BLA of conditional KO mice compared to their littermates. PFC: Mann–Whitney test, *P* = 0.0159, *Hdac2*^*lox/lox*^ *n* = 4; *PV-Cre; Hdac2*^*lox/lox*^ *n* = 5. BLA: Mann–Whitney test, *P* = 0.0140*, Hdac2*^*lox/lox*^ *n* = 7; *PV-Cre; Hdac2*^*lox/lox*^ *n* = 6). **d** Coronal sections of PFC of *PV-Cre; Hdac2*^*lox/lox*^ and control littermates labeled with WFA (PNN, cyan) and PV (magenta). White arrows indicate PV-positive cells surrounded by PNN (PV^+^PNN^+^ cell bodies), while yellow arrows point to PV-positives cell that are not surrounded by PNN (PV^+^PNN^-^ cell bodies). Scale bar, 40 µm. **e** The percentage of PV^+^ cell bodies surrounded by WFA-stained PNN is significantly reduced in the PFC and BLA, but not in the SSCX, of conditional KO mice compared to their littermates. PFC: Mann–Whitney test, *P* = 0.0173, *Hdac2*^*lox/lox*^, *n* = 6; *PV-Cre; Hdac2*^*lox/lox*^, *n* = 5. BLA: Mann–Whitney test, *P* = 0.0087, *Hdac2*^*lox/lox*^, *n* = 6; *PV-Cre; Hdac2*^*lox/lox*^, *n* = 6. SSCX: Mann–Whitney test, *P* = 0.6753, *Hdac2*^*lox/lox*^, *n* = 6; *PV-Cre; Hdac2*^*lox/lox*^ *n* = 6. Data represent mean ± s.e.m. Black circle represent individual data points. *P < 0.05, **P < 0.01.

During their maturation process, PV^+^ cells form numerous perisomatic synapses on pyramidal cell somata over the first 5 postnatal weeks in rodent neocortex [37]. To investigate whether *Hdac2* conditional deletion in PV^+^ cells affected their efferent connectivity, we quantified putative perisomatic synapses formed by PV^+^ cells onto pyramidal cells by co-immunolabeling with gephyrin (scaffolding protein associated with GABA-A receptor, postsynaptic) and PV, in PFC and BLA. We found that the density of PV^+^ boutons colocalizing with gephyrin was significantly reduced in both PFC and BLA of naive conditional KO mice compared to their control littermates (Fig. 2a, b). This difference was not due to decreased PV expression, since the density of perisomatic PV^+^ boutons was not significantly different between the two genotypes (Fig. 2b).

Previous reports showed that remodeling of perisomatic inhibitory synapses was critical for efficient extinction of fear memories [15, 17]. To investigate whether PV^+^ cell synapse remodeling was affected by *Hdac2* deletion, we analyzed PV^+^ cell perisomatic synapses in both PFC and BLA 24 h after the end of fear extinction training in *PV-Cre; Hdac2*^*lox/lox*^ mice and their control littermates. While naive conditional mutant mice showed decreased percentage of perisomatic PV^+^ boutons colocalizing with gephyrin puncta compared to control mice in both PFC and BLA (Fig. 2c, Supplementary Fig. 3a, b, e), no significant difference could be detected between the two genotypes following extinction training (Supplementary Fig. 3c, d, e). These results suggest that fear extinction training promoted more extensive PV^+^ cell synapse remodeling in *PV-Cre; Hdac2*^*lox/lox*^ mice than in control littermates, reinforcing the hypothesis that changes in PV^+^ cell-mediated inhibition is critical for reducing the spontaneous recovery of fear memory over time [15].

### Postnatal deletion of *Hdac2* in PV^+^ cells reduces PNN aggregation around their somata

PNN appearance around PV^+^ cell somata and dendrites is developmentally regulated, and correlates with increased inhibition and critical period closure in sensory cortices [38]. PV^+^ cell enwrapping by PNN has been shown to be a limiting factor for adult brain plasticity [20, 29, 30]. In particular, chondroitin sulfate proteoglycans assembly into PNNs promotes the formation of extinction-resistant memories [28]. Since we observed reduced spontaneous recovery of fear memory over time following extinction training in adult *PV-Cre*; *Hdac2*^*lox/lox*^ mice, we reasoned that this process may be accompanied by PNN reduction in conditional KO mice. Using WFA staining to label PNNs, we indeed found a significant decrease in the percentage of PV^+^ cells surrounded by PNNs in PFC and BLA, two regions directly implicated in fear behavior regulation, in naive mutant mice compared to control littermates (Fig. 2c, d). Of note, we observed that PNNs were not localized exclusively around PV^+^ cells in the BLA (data not shown), consistent with previous reports [39, 40]. Unexpectedly, we did not observe a significant difference in the percentage of PNN^+^PV^+^ cells in the SSCX of *PV-Cre*; *Hdac2*^*lox/lox*^ compared to *Hdac2*^*lox*^ mice (Fig. 2d), suggesting that epigenetic regulation of PNN organization is region-specific in the adult mouse cortex.

### Hdac2 regulates Aggrecan expression in PV^+^ cells

**Fig. 3:**
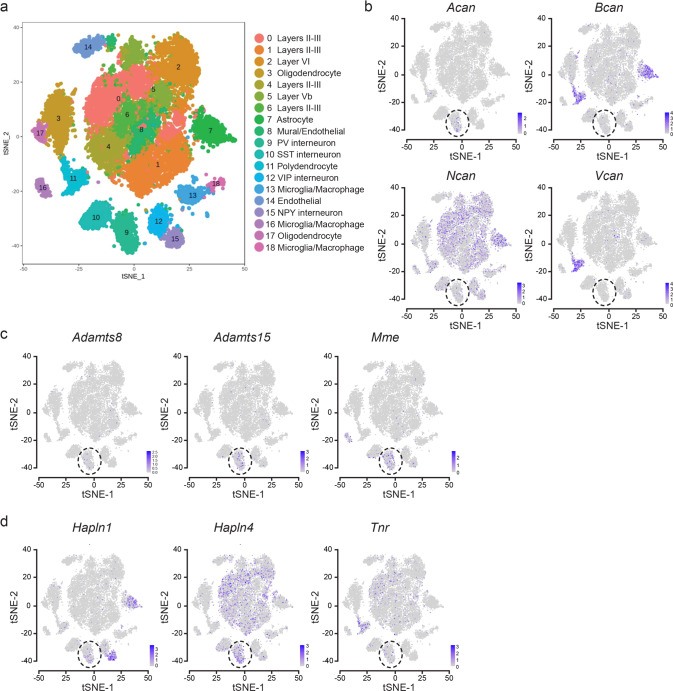
PNN components and metalloproteases are expressed by different cell types in mouse PFC. **a** Single cell RNA-sequencing (DropSeq) of PFC cells of P40-43 wild-type mice (*n* = 8 mice). t-SNE visualization of identified cell types; cluster 9 refers to PV^+^ cells. **b**–**d** Expression localization of critical components of PNN structure and remodeling, including lecticans (**b** *Acan*, *Bcan*, *Ncan*, and *Vcan*), metalloproteases known to be expressed by PV^+^ cells (**c** *Adamts8*, *Adamts15*, and *Mme*) and other critical PNN components (**d** *Halpn1*, *Halpn4*, and *Tnr*).

WFA specifically binds to CAG-chains [41] in all lecticans. Since we observed decreased WFA labeling surrounding PV^+^ cell somata in brain regions involved in fear regulation in *PV-Cre*; *Hdac2*^*lox/lox*^ mice (Fig. 2c, d), we next sought to identify which lecticans are cell-autonomously expressed by PV^+^ cells, and could thus be directly affected by PV^+^ cell-specific *Hdac2* deletion. Using Drop-Seq, we transcriptionally profiled PFC cells from 6 weeks-old wild-type mice. Samples from 5 biological replicates yielded 19393 cells that were sequenced to a median depth of 15,555 aligned reads/cell (representing a median of 1427 genes/cell) and controlled for quality (Supplementary Fig. 4). Cells were then clustered and represented in t-Distributed Stochastic Neighbor Embedding (t-SNE) plots (Fig. 3a). Cell clusters were defined as glutamatergic neurons, GABAergic neurons or non-neuronal cells based on *Slc17a7*, *Gad1* and *Gad2* detection (Supplementary Fig. 5). Using the Dropviz database as a template for murine cortical transcriptomes, clusters were then associated with well-known neuronal and non-neuronal cell groups. Among the latter, we distinguished astrocytes, oligodendrocytes/polydendrocytes, microglial cells/macrophages as well as endothelial cells. Within neuronal cell types, VGLUT^+^ glutamatergic neurons and Gad1^+^/Gad2^+^ interneurons were identified, including PV-expressing interneurons (cluster 9, Supplementary Fig. 6).

Next, we analyzed which cell types express mRNAs coding for known PNN structural components and remodeling enzymes (metalloproteases). We confirmed that the metalloproteases *Adamts8*, *Adamts15*, and *Mme* were highly enriched in PV^+^ cells, (Fig. 3c and Supplementary Fig. 6), as previously reported [42, 43]. Of the 4 analyzed lecticans, *Neurocan* was the most abundant and was expressed by different neuronal cell types and astrocytes. *Brevican* was also expressed by different cell types, including PV^+^ cells, but it was highly enriched in astrocytes and in CPSG4-expressing NG2^+^ polydendrocytes. The latter were also the major source of *Versican* (Fig. 3b and Supplementary Fig. 6). *Acan*, on the other hand, was mostly expressed by PV^+^ cells, and to a much lesser extent, by polydendrocytes (Fig. 3b and Supplementary Fig. 6). None of the other PNN components analyzed (*Hapln1*, *Hapln4*, *Tnr*) showed cell type-specific expression (Fig. 3d and Supplementary Fig. 6).

**Fig. 4:**
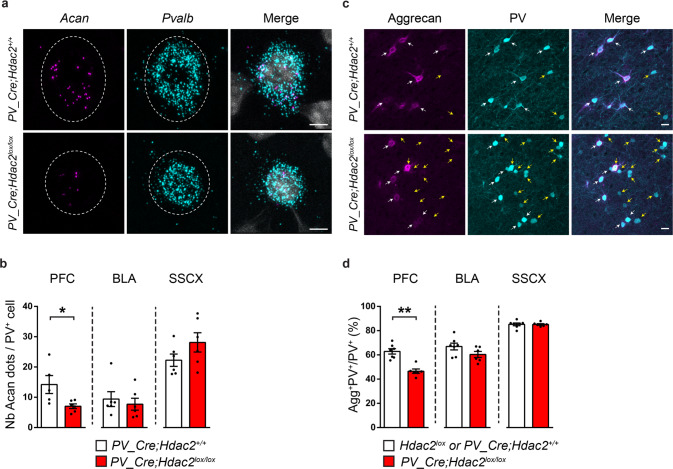
PV^+^ cell-specific *Hdac2* deletion leads to reduced *Acan* mRNA expression and Aggrecan condensation around PFC PV^+^ cell somata. **a** Representative images of fluorescent RNAscope in situ hybridization against *Acan* (Aggrecan, magenta) and *Pvalb* (PV, cyan) in prefrontal cortex of *PV-Cre; Hdac2*^*lox/lox*^ and control littermates (DAPI labeling is in gray). Somata expressing *Pvalb* mRNA are outlined. Scale bar, 5 µm. **b** The mean number of *Acan* dots detected in *Pvalb*^+^ cells is significantly lower in the PFC, but not in BLA or SSCX, of conditional knockout mice compared to their control littermates. PFC: Mann–Whitney test, *P* = 0.0173, *PV-Cre; Hdac2*^*+/+*^ *n* = 5, *PV-Cre;Hdac2*^*lox/lox*^ *n* = 6. BLA: Mann–Whitney test*, P* = 0.3874, *PV-Cre; Hdac2*^*+/+*^ *n* = 6, *PV-Cre;Hdac2*^*lox/lox*^ *n* = 6. SSCX: Mann–Whitney test, *P* = 0.1797, *PV-Cre; Hdac2*^*+/+*^ *n* = 6, *PV-Cre; Hdac2*^*lox/lox*^ *n* = 6. **c** Prefrontal cortex coronal sections labeled for Aggrecan (cyan) and PV (magenta) in *PV-Cre; Hdac2*^*lox/lox*^ and control littermates. White arrows indicate PV-positive cells surrounded by Aggrecan (PV^+^Aggrecan^+^ cell bodies), while yellow arrows indicate PV-positive cells that are not surrounded by Aggrecan (PV^+^Aggrecan^−^ cell bodies). Scale bar, 10 µm. **d** The percentage of PV^+^ cell bodies surrounded by aggrecan is significantly reduced in the PFC, but not BLA or SSCX, of conditional knockout mice compared to their control littermates. PFC: Mann–Whitney test, *P* = 0.0012, BLA: Mann–Whitney test *P* = 0.0513, SSCX: Mann–Whitney test *P* = 0.6037. For all three brain regions, *Hdac2*^*lox/lox*^ or *PV-Cre; Hdac2*^*+/+*^ *n* = 7; *PV-Cre; Hdac2*^*lox/lox*^ *n* = 6. Data represent mean ± s.e.m. Black circle represent individual data points. *P < 0.05, **P < 0.01.

In particular, Aggrecan, coded by *Acan*, plays an obligatory role in PNN formation. Indeed, *Acan* downregulation leads to the disappearance of PNN aggregation around PV^+^ cells, both in brain-wide homozygous *Acan* mutant mice and following virally-induced *Acan* deletion [27]. To confirm the Drop-Seq finding and further investigate whether *Acan* expression is regulated by Hdac2 in PV^+^ cells, we analyzed *Acan* transcripts by RNAscope hybridization in situ in PFC, BLA and SSCX of adult *PV-Cre*; *Hdac2*^*lox/lox*^ mice and control littermates (Fig. 4). We confirmed that a majority of PFC PV^+^ cells expressed *Acan* in wildtype mice (percentage of *PV*^*+*^*Acan*^*+*^/*PV*^*+*^ cells = 71.4 ± 3.6%, *n* = 7 mice). We further found that PFC PV^+^ cells expressing detectable level of *Acan* mRNA expressed also higher levels of *Pvalb* mRNA compared to PV^+^ cells negative for *Acan* (Supplementary Fig. 7).

Interestingly, in *PV-Cre*; *Hdac2*^*lox/lox*^ mice, *Acan* expression was significantly reduced specifically in the PFC, but not in the SSCX or the BLA, compared to control littermates (Fig. 4a, b). This decrease in *Acan* expression was accompanied by a significant decrease in the percentage of PV^+^ cells surrounded by Aggrecan immunostaining specifically in the PFC (Fig. 4c, d). Unaltered Aggrecan expression levels in SSCX of mutant mice is consistent with the observation that the percentage of PNN^+^PV^+^ cells in the SSCX was not affected (Fig. 2c, d). Conversely, the percentage of PNN^+^PV^+^ cells was reduced in the BLA in absence of parallel changes in the percentage of Aggrecan^+^PV^+^ cells (Fig. 2c, d; Fig. 4). Taken together, these data strongly point towards a strict requirement for Aggrecan in PNN formation specifically around prefrontal PV^+^ cells.

**Fig. 5:**
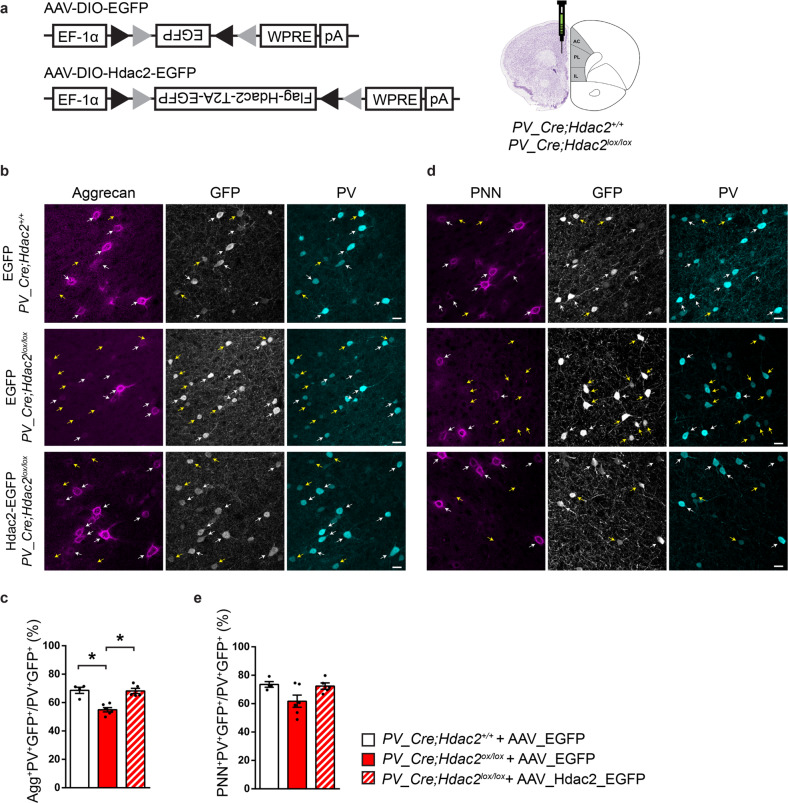
PV^+^ cell-specific Hdac2 re-expression in *PV_Cre; Hdac2*^*lox/lox*^ mice rescues Aggrecan condensation around PFC PV^+^ cell somata. **a** Schematic representation of Cre-dependent viral vectors and experimental approach. **b** Immunolabelling of Aggrecan (magenta), GFP (gray) and PV (cyan) in PFC coronal sections from *PV-Cre; Hdac2*^*+/+*^ and *PV-Cre; Hdac2*^*lox/lox*^ mice injected with the control AAV (AAV_DIO_EGFP) and from *PV-Cre; Hdac2*^*lox/lox*^ mice injected with the Cre-dependent Hdac2 expressing virus (AAV_DIO_Hdac2_T2A_EGFP). White arrows point to the somata of AAV-transfected (GFP-positive) PV cells surrounded by Aggrecan (PV^+^GFP^+^Aggrecan^+^ cell bodies), while yellow arrows indicate AAV-transfected (GFP-positive) PV cells that are not surrounded by Aggrecan (PV^+^GFP^+^Aggrecan^−^ cell bodies). Scale bar, 10 µm. **c** Percentage of PV^+^GFP^+^ cell bodies surrounded by Aggrecan. Kruskall–Wallis with Dunn’s *posthoc* test, *P* = 0.0008. **d** Labeling for WFA (PNN, magenta), GFP (gray) and PV (cyan) in the three experimental groups. White arrows indicate PV^+^GFP^+^ cell bodies enwrapped by PNN (PV^+^GFP^+^PNN^+^ somata), while yellow arrows indicate PV^+^GFP^+^ cell bodies that are not enwrapped by PNN (PV^+^GFP^+^PNN^−^ somata). Scale bar, 10 µm. **e** Percentage of PV^+^GFP^+^ cell bodies surrounded by WFA-stained PNN in the prefrontal cortex. Kruskall–Wallis with Dunn’s *posthoc* test, *P* = 0.2603. Number of mice: *PV-Cre; Hdac2*^*+/+*^ + AAV_DIO_EGFP, *n* = 4, *PV-Cre; Hdac2*^*lox/lox*^ + AAV_DIO_EGFP, *n* = 6, *PV-Cre; Hdac2*^*lox/lox*^ + AAV_DIO_Hdac2_T2A_EGFP, *n* = 5. Data represent mean ± s.e.m. Black circle represent individual data points. *P < 0.05.

The effects of PV^+^ cell-specific *Hdac2* deletion on PNN aggregation can be due to a direct regulation of the expression of critical PNN components by Hdac2, or due to secondary homeostatic effects caused by long-term *Hdac2* deletion. To test whether Hdac2 regulates Aggrecan expression in PV^+^ cells, we virally re-expressed Hdac2 with EGFP or EGFP alone specifically in PFC PV^+^ cells of adult *PV-Cre*; *Hdac2*^*lox/lox*^ and *PV_Cre*; *Hdac2*^*+/+*^ mice for three weeks using an inverted double-floxed *Hdac2* coding region driven by a ubiquitous EF-1α promoter (Fig. 5a). Consistent with our previous data (Fig. 4d), we observed a significant reduction in the percentage of PV^+^ cells enwrapped by Aggrecan in conditional KO compared to wildtype littermates when both were injected with the control virus (AAV_DIO_EGFP; Fig. 5b, c). On the other hand, injection of AAV_DIO_Hdac2-T2A-EGFP was sufficient to rescue the percentage of Aggrecan^+^ PV^+^ cells (Fig. 5c) and to increase (albeit not significantly) the number of PV^+^ cells surrounded by WFA-stained PNN in *PV-Cre*; *Hdac2*^*lox/lox*^ mice (Fig. 5d, e).

**Fig. 6:**
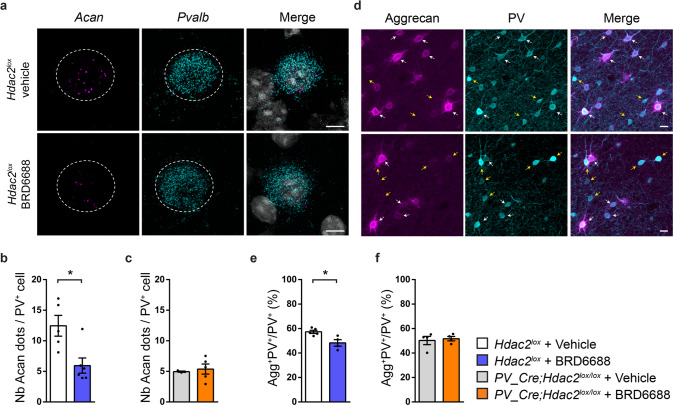
Hdac2 inhibition decreases *Acan* mRNA expression and Aggrecan agglomeration around PFC PV^+^ cell somata in wild-type but not in *PV-Cre; Hdac2*^*lox/lox*^ mice. **a** Representative images of fluorescent RNAscope in situ against *Acan* (Aggrecan, magenta) and *Pvalb* (PV, cyan) around a DAPI (gray) nucleus in PFC of *Hdac2*^*lox/lox*^ mice 17 h after i.p. injection of either the Hdac2 inhibitor BRD6688 or the vehicle solution. Somata expressing *Pvalb* mRNA are outlined. Scale bar, 5 µm. **b** The mean number of *Acan* dots present around a *Pvalb*^+^ cell is reduced in BRD6688-treated wild-type (Mann–Whitney test, *P* = 0.0303) (**c**) but not conditional knockout mice (Mann–Whitney test, *P* = 0.6786). Number of *Hdac2*^*lox/lox*^ injected with vehicle, *n* = 5 or BRD6688, *n* = 6. Number of *PV-Cre; Hdac2*^*lox/lox*^ mice injected with vehicle *n* = 3 or BRD6688, *n* = 5. **d** PFC coronal sections immunolabeled for Aggrecan (cyan) and PV (magenta) in *Hdac2*^*lox/lox*^ mice 17 h after i.p. injection of either the Hdac2 inhibitor BRD6688 or the vehicle. White arrows indicate PV-positive cells surrounded by Aggrecan (PV^+^Aggrecan^+^ cell bodies), while yellow arrows indicate PV-positive cells that are not surrounded by Aggrecan (PV^+^Aggrecan^−^ cell bodies). Scale bar, 10 µm. **e** The percentage of PV^+^ cell bodies surrounded by Aggrecan is reduced by BDR6688 injection in control (Mann–Whitney test, *P* = 0.0317) (**f**) but not conditional knockout mice (Mann–Whitney test, *P* = 0.8286). Number of *Hdac2*^*lox/lox*^ injected with vehicle, *n* = 5 or BRD6688, *n* = 4. Number of *PV-Cre;Hdac2*^*lox/lox*^ mice injected with vehicle, *n* = 4 or BRD6688, *n* = 4. Graph bars represent mean ± s.e.m. Circles represent individual mouse values. *P < 0.05.

To further support the hypothesis that Hdac2 activity in PV^+^ cells regulates Aggrecan levels, we explored the effects of acute Hdac2 pharmacological inhibition. We found a significant decrease in the number of *Acan* mRNA molecules in PFC PV^+^ cell somata (Fig. 6a, b) and in the proportion of PV^+^ cells enwrapped by Aggrecan^+^ nets (Fig. 6d, e) 17 h after BRD6688 i.p. injection in wild-type mice. Conversely, the same treatment was unable to further reduce *Acan*/Aggrecan expression in *PV-Cre; Hdac2*^*lox/lox*^ mice (Fig. 6c, f), suggesting that the effect of BRD6688 treatment was mostly mediated by Hdac2 expression in PV^+^ cells,

Overall, these results show that PV^+^ cells are the major source of Aggrecan in the PFC and that Hdac2 modulates PNN aggregation by regulating *Acan* transcription in PV^+^ cells.

### Reduced *Hdac2* activity in PFC PV^+^ cells promotes dynamic changes in *Acan* expression following extinction training

**Fig. 7:**
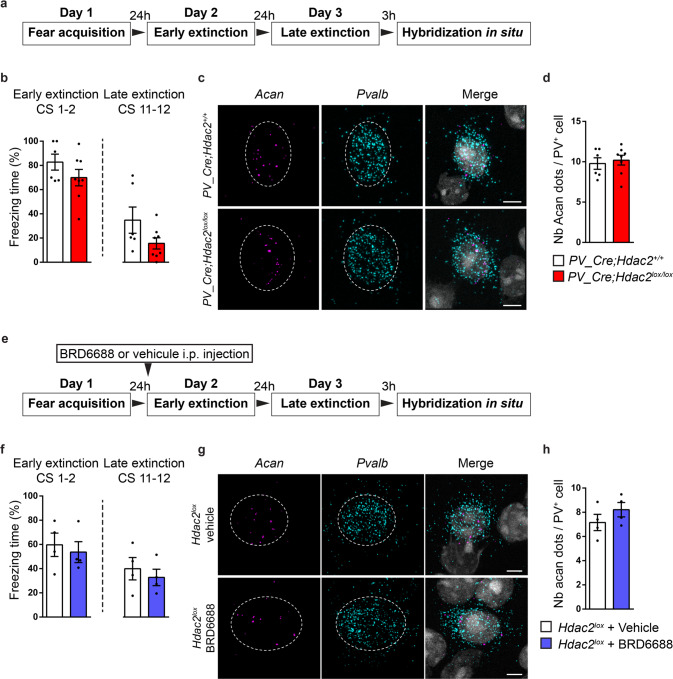
*Acan* expression by PFC PV^+^ cells at the end of extinction training does not differ in conditional HDAC2 KO mice or in BRD6688-injected wild-type mice compared to controls. **a** Schematic representation of the experimental protocol. Brains of *PV-Cre; Hdac2*^*lox/lox*^ mice and their control littermates were collected 3 h after late extinction training for in situ hybridization. **b** Both *PV-Cre; Hdac2*^*lox/lox*^ mice and controls show efficient fear extinction. Repeated two-way ANOVA; F_genotype_ (1,12) = 7.554, *P* = 0.0177, F_extinction_(11,132) = 9.148, *P* < 0.0001, F_genotype_*_extinction_(11,132) = 1.107, *P* = 0.3605. **c** Representative images of fluorescent RNAscope in situ against *Acan* (Aggrecan, magenta) and *Pvalb* (PV, cyan) around a DAPI (gray) nucleus in PFC of *PV-Cre; Hdac2*^*+/+*^ and *PV-Cre; Hdac2*^*lox/lox*^ mice 3 h after late extinction training. Somata expressing *Pvalb* mRNA are outlined. Scale bar, 5 µm. **d** The mean number of *Acan* dots present around a prefrontal *Pvalb*^+^ cell 3 h after late extinction training is comparable between *PV-Cre; Hdac2*^*+/+*^ and *PV-Cre; Hdac2*^*lox/lox*^ mice (Mann–Whitney test, *P* = 0.8112). Number of mice: *PV-Cre; Hdac2*^*+/+*^ *n* = 6; *PV-Cre; Hdac2*^*lox/lox*^ *n* = 8. **e** Schematic representation of the experimental protocol. Brains of BRD6688-injected or vehicle-injected mice 6 h before extinction training have been collected 3 h after late extinction training for in situ hybridization. **f** Both BRD6688-injected and vehicle-injected controls show efficient fear extinction. Repeated two-way ANOVA; F_treatment_ (1,6) = 1.093, *P* = 0.3362, F_extinction_(1166) = 3.902, *P* = 0.0002, F_treatment_*_extinction_ (1166) = 0.7446, *P* = 0.6922. **g** Representative images of fluorescent RNAscope in situ against *Acan* (Aggrecan, magenta) and *Pvalb* (PV, cyan) around a DAPI (gray) nucleus in PFC of BRD6688-injected and vehicle-injected mice 3 h after late extinction training. Somata expressing *Pvalb* mRNA are outlined. Scale bar, 5 µm. **h** The mean number of *Acan* dots present around a prefrontal *Pvalb*^+^ cell 3 h after late extinction training is comparable between BRD6688-injected and vehicle-injected mice (Mann–Whitney test, *P* = 0.2). Number of mice: vehicle-injected *n* = 4; BRD6688-injected *n* = 4.

Class 1 Hdac (including Hdac2) inhibition has been shown to prime the expression of neuroplasticity-related genes [23]. We thus asked whether *Acan* expression might be more dynamic in *Hdac2*^*−/−*^ PV^+^ cells following extinction training. To answer this question, we collected brains from *PV-Cre; Hdac2*^*lox/lox*^ mice and control littermates 3 h after the end of extinction training (Fig. 7a, b) and quantified *Acan* mRNA expression in PV^+^ cells from the PFC (Fig. 7c, d). We found that, although *Acan* expression was reduced in PFC PV^+^ cells from naive mutant mice compared to wild-type littermates (Fig. 4a, b), its expression reached wild-type levels after extinction training (Fig. 7c, d). Next, we asked whether acute pharmacological Hdac2 inhibition could promote *Acan* dynamic changes triggered by extinction learning. While naive mice injected with BRD6688 showed reduced *Acan* levels (Fig. 6a, b), mice injected with BRD6688 following fear acquisition but before the onset of extinction training showed no significant difference between *Acan* levels compared to vehicle-treated controls 3 h after the end of extinction training (Fig. 7g, h).

Altogether, these results indicate that either PV^+^ cell-specific Hdac2 deletion or pharmacological inhibition allows for dynamic changes in *Acan* expression following extinction training.

### *Acan* knock-down during extinction training is sufficient to limit the spontaneous recovery of fear memories with time

**Fig. 8:**
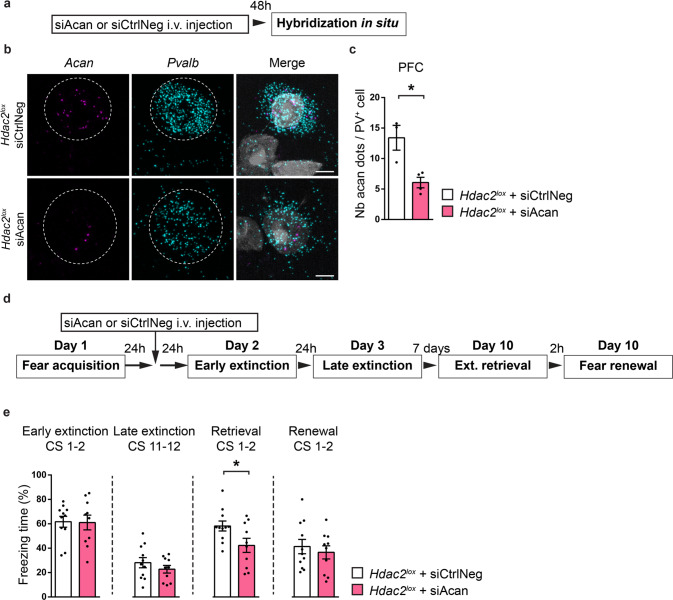
*Acan* knock-down during extinction training is sufficient to increase the persistence of extinction memories. **a** Schematic representation of the experimental protocol. Brains of adult *Hdac2*^*lox*^ mice were collected 48 h after intravenous injection (i.v.) of either TARBP-BTP:siCtrlNeg or TARBP-BTP:siAcan. **b** Representative images of fluorescent RNAscope in situ against *Acan* (Aggrecan, magenta) and *Pvalb* (PV, cyan) around a DAPI (gray) nucleus in PFC of *Hdac2*^*lox*^ mice 48 h after the i.v. injection. Somata expressing *Pvalb* mRNA are outlined. Scale bar, 5 µm. **c** The mean number of *Acan* dots in PFC *Pvalb*^+^ cells 48 h after injection of TARBP-BTP:siAcan is significantly decreased compared to those in PFC *Pvalb*^+^ cells from TARBP-BTP:siCtrlNeg injected mice (Unpaired two tailed t-test, *P* = 0.0139). Number of mice: *Hdac2*^*lox*^ + TARBP-BTP:siCtrlNeg *n* = 3; *Hdac2*^*lox*^ + TARBP-BTP:siAcan *n* = 4. **d** Schematic representation of the experimental protocol. Ext. extinction. **e** TARBP-BTP:siCtrlNeg-injected and TARBP-BTP:siAcan-injected mice show similar fear extinction rate. Repeated two-way ANOVA; F_treatment_ (1,19) = 1.109, *P* = 0.3054, F_extinction_(11,209) = 18.85, *P* < 0.0001, F_treatment_*_extinction_ (11,209) = 1.052, *P* = 0.4021. One week after extinction training, freezing levels in the retrieval test are significantly lower in TARBP-BTP:siCtrlNeg-injected compared to TARBP-BTP:siAcan-injected mice. Extinction retrieval unpaired two-tailed *t*-test, *P* = 0.0349; fear renewal, unpaired two-tailed *t*-test, *P* = 0.5608. Number of *Hdac2*^*lox*^ mice injected with TARBP-BTP:siCtrlNeg, *n* = 11 or TARBP-BTP-siAcan, *n* = 10. Graph bars represent mean ± s.e.m. Circles represent individual mouse values. CS conditioned stimulus. *P < 0.05.

Finally, we asked whether the observed dynamic changes in *Acan* levels caused by Hdac2 inhibition were sufficient to attenuate the spontaneous recovery of fear memories following extinction training over time. To answer this question, we sought to transiently decrease *Acan* expression after fear acquisition but before extinction training. To this purpose, we used a chimeric fusion protein comprising a double-stranded RNA binding domain fused to a brain targeting peptide, namely TARBP-BTP, to deliver siRNA to the brain [44, 45]. Previous data showed that using TARBP-BTP fusion protein as a carrier facilitated siRNA delivery across the brain-blood barrier to the brain [44]. We observed a significantly reduced number of *Acan* mRNA molecules in PFC PV^+^ cells compared to the control group 48 h after TARBP-BTP/*Acan*-siRNA intravenous injection in wild-type adult mice (Fig. 8a–c), demonstrating that siRNAs were efficiently delivered to the brain parenchyma and could enter PV^+^ cells. We then injected either TARBP-BTP/*Acan-*siRNA or TARBP-BTP/*CtrlNeg*-siRNA in wild-type mice, 24 h after fear learning, and then subjected them to fear extinction training 24 h after injection (Fig. 8d). Compared to control TARBP-BTP/*CtrlNeg*-siRNA mice, TARBP-BTP/*Acan-*siRNA-injected mice presented significantly less freezing behavior during the retrieval test (Fig. 8e). Therefore, *Acan* knock-down in PV^+^ cells prior to extinction training was sufficient to render extinction memories stronger and limits the spontaneous recovery of fear memories with time.

## Discussion

The maturation of GABAergic circuits, in particular of PV^+^ cells, is one of the factors that restricts critical period plasticity in the adult brain [11, 12, 38]. Hence, controlled manipulation of cortical PV^+^ cell function can reinstate heightened plasticity, creating an opportunity for therapeutic intervention [46, 47]. To this purpose, it is essential to gain a better understanding of the molecular mechanisms determining PV^+^ cell maturation state in the adult brain. Here, we showed that the epigenetic landscape of PV^+^ cells regulates their maturation state, thereby affecting adult neuroplasticity. In particular, our results demonstrate that PV^+^ cell-specific *Hdac2* deletion reduces PNN aggregation around PV^+^ cell somata and PV^+^ cell synapse density in naive mice, while promoting PV^+^ cell structural synapse remodeling following extinction training. We further found that Hdac2 levels controlled the expression of the lectican Aggrecan, a critical regulator of PNN aggregation, by regulating its transcription specifically in PFC PV^+^ cells. These anatomical changes are accompanied by decreased spontaneous recovery of fear memories with time in mutant mice. Finally, siRNA-mediated brain-targeted *Acan* reduction after fear acquisition coupled with extinction training was sufficient to reduce the spontaneous recovery of fear memory with time in adult wild-type mice, indicating a potential therapeutic target for enhancing extinction-dependent plasticity.

A previous study by Gogolla and collaborators [28] showed that degradation of PNNs by chondroitinase ABC injection in adult BLA rendered subsequently acquired fear memories susceptible to erasure following extinction learning. However, the same treatment had no effect when applied after fear learning, but before extinction training. In contrast, our data showed that global *Acan* reduction after fear learning was sufficient to reduce the spontaneous recovery of fear memories one week after extinction training. It is possible that PNN remodeling in PFC and not, or not exclusively, in the BLA, is essential for long term retention of extinction learning. Alternatively, the dynamic regulation of PNN expression, more than its absolute levels, may play a key role in this process. Indeed, a transient decrease in *Acan* expression after fear learning but before extinction training attenuated the spontaneous recovery of fear memory over time, suggesting that dynamic changes of PNNs could be a critical step in this process. To test this hypothesis, we choose to deliver siRNA against *Acan* using a peptidic carrier that crosses the brain blood barrier Weather transient *Acan* changes and PNN remodeling selectively in PFC are sufficient to affect the spontaneous recovery of fear memory, or whether PNN remodeling needs to occur in the BLA too, remain to be established.

Numerous studies have addressed the role of epigenetic modifiers, through pharmacological inhibition or genetic deletion of different Hdacs, on fear memory formation and extinction [48]. In particular, an elegant study demonstrated that pharmacological inhibition of Hdac2 during contextual fear extinction prevents the spontaneous recovery of freezing behavior during the recall of remote fear memories by upregulating neuroplasticity-related gene expression [23]. However, whether Hdac2 plays a different role in different neuron types thus leading to distinct behavioral outputs, is not clear. A previous study showed that *Hdac2* deletion in post-mitotic forebrain glutamatergic neurons (using CaMKII-Cre mice) led to a robust acceleration of extinction learning, which was however followed by a normal spontaneous recovery of fear memories 5 days after the end of the extinction [26]. Conversely, our data showed that *Hdac2* deletion specifically in postnatal PV^+^ cells did not affect the rate of extinction learning but led to decreased spontaneous recovery of fear memories with time. The specific role of Hdac2 expressed by PV^+^ cells in allowing spontaneous fear recovery was further supported by the fact that the beneficial effect of Hdac2 inhibitor and PV^+^ cell-specific deletion of *Hdac2* on preventing the recovery of fear memories were not additive. These different results could be explained by partly different roles played by glutamatergic and PV^+^ neurons in fear extinction learning and spontaneous fear memory recovery. It is also conceivable that Hdac2 regulates a different set of genes in different cell types, thus leading to different cellular responses.

The main problem with fear extinction is the reappearance, or spontaneous recovery, of the original fear after extinction training. To explain this phenomena, two opposite models of extinction have been proposed and strongly debated: one posits that the extinction training leads to the erasure of the fear memory itself [17, 28], while the second proposes that extinction creates new inhibitory learning, which controls the fear memory [49, 50]. It has been suggested that extinction learning could be seen as an equilibrium between erasure and inhibition, where any behavioral manipulation which influences fear expression would either promote a functional erasure of the original fear trace or have permissive effects on the new inhibitory memory [51]. In the field of cognitive neuro-epigenetics, it has been proposed that “there exists an equilibrium in epigenetic states that can be pushed in one direction or another” [51]. The data presented in this work support the hypothesis that increasing chromatin acetylation, likely at specific genes, enhances the retention of fear extinction memories over time. In particular, we identified PV^+^ cell-epigenetic state as a major determinant in the spontaneous recovery of fear memories over time. Whether Hdac2 inhibition in PV^+^ cells leads to stronger inhibitory memory opposing the fear memory, or to the erasure of the original fear memory is not clear. However, the lack of generalization of fear extinction memory, as observed by similar freezing times during the renewal test in *PV-Cre;Hdac2*^*lox/lox*^ versus *PV-Cre;Hdac2*^*+/+*^ mice (Supplementary Fig. 2) or following Hdac2 inhibition after fear memory acquisition (Fig. 1g), argues against the erasure of the original fear memory, instead supporting the hypothesis that PV^+^ cell heightened plastic remodeling could contribute to forming stronger (or more stable) inhibitory memory [15].

The development of cortical PV^+^ cells is a prolonged process which plateaus only by the end of adolescence [37]. During the postnatal maturation phase, cortical PV^+^ cells form exuberant innervation fields characterized by baskets of perisomatic synapses. In addition, during their maturation phase, PV^+^ cell somata and dendrites are progressively enwrapped in PNNs, thought to stabilize their synaptic inputs [19, 52] and protect them from oxidative stress [53]. Our data suggest that PV^+^ cell-epigenetic modifications likely regulate their maturation state, since *PV-Cre;Hdac2*^*lox/lox*^ mice showed significantly reduced PNN condensation around PV^+^ cells and decreased density of PV^+^ cell perisomatic synapses in the PFC and BLA, two brain regions implicated in fear behavior regulation. Whether these structural changes in PV^+^ cells synaptic density correlate with reduced PV^+^ cell-mediated functional inhibition in the BLA or/and PFC remains to be determined. Nevertheless, our data is consistent with the observation that PV^+^ cell-restricted *Hdac2* deletion reduced evoked inhibitory responses and enhanced long-term depression in the visual cortex of adult mice, which are phenotypes typically associated with younger mice [27].

We further found that PFC PV^+^ cells expressing detectable level of *Acan* mRNA also expressed higher levels of *Pvalb* mRNA compared to PV^+^ cells negative for *Acan* (Supplementary Fig. 7). Higher number of PV^+^ cells expressing high levels of PV protein have been associated with reduced memory and structural synaptic plasticity in the hippocampus [18] and visual cortex [13]. This observation supports the hypothesis that reduced *Acan* expression in the PFC of conditional KO mice (Fig.4) or in wild-type mice injected with BRD6688 (Fig.6) may lead to a PV^+^ cell network configuration favoring plasticity. In accordance with this hypothesis, Chen et al. [14] showed that extinction learning increased the fraction of PV^+^ cells expressing low PV levels, which are thought to be more plastic [18]. They further showed that chemogenetic-mediated manipulations of PV^+^ cell activity affected mouse freezing during extinction learning, with more active PV^+^ cells leading to higher freezing. However, this study did not explore whether and how these manipulations affected long-term spontaneous recovery of fear memories.

Trouche et al. [15] showed that extinction training induced structural remodeling of perisomatic PV^+^ cell synapses specifically around excitatory neurons that had been previously activated during the encoding of the original fear memory (fear neurons) in the BLA. In addition, Bhagat et al. [17] reported that PV^+^ cell-specific deletion of Nogo Receptor 1 enhanced BLA PV^+^ cell synaptic structural remodeling and reduced spontaneous recovery of fear memory over time. In *PV-Cre;Hdac2*^*lox/lox*^ mice, we observed increased remodeling of PV^+^ cell synapses in both BLA and PFC following extinction training. Elegant work demonstrated that PFC PV^+^ cell firing inversely correlates with freezing behavior and that their optogenetic activation reduces freezing behavior [54]. Altogether, this data support the hypothesis that reinforcing PFC PV^+^ cell-mediated inhibitory drive by the end of extinction training could contribute to the maintenance of the extinction memory, thereby leading to reduced fear response freezing at the retrieval test.

How and to what extent PV^+^ cells control the development, integrity and dynamic of the PNNs surrounding their somata and dendrites, in a cell-autonomous fashion, is still an open question. By using single-cell transcriptomic analysis, we found that PFC PV^+^ cells express a variety of PNN molecular components and metalloproteases. While most of these factors were expressed by other cell types as well, *Acan* transcription appeared to be mostly restricted to PV^+^ cells, consistent with previous studies [55, 56]. In vitro [57, 58] and in vivo [30] experiments revealed that Aggrecan, coded by *Acan*, plays a critical role in PNN formation. For instance, brain-wide targeting of *Acan* (*Nes_Cre; Acan*^*lox/lox*^ mice) or virally-mediated local *Acan* deletion in adult visual cortex led to complete loss of WFA-stained PNN, while conditional deletion of one *Acan* allele was sufficient to reduce WFA staining intensity [30]. Therefore, the reduced Aggrecan levels we observed in *PV_Cre; Hdac2*^*lox/lox*^ or BRD6688-injected mice likely contributes to impaired aggregation of other extracellular matrix molecules into PNNs. Our data showed that PV^+^ cell-specific *Hdac2* deletion reduced *Acan/*Aggrecan expression levels in PV^+^ cells, while re-introduction of Hdac2 in *Hdac2*^*−/−*^ mutant cells rescued *Acan/*Aggrecan expression levels, thus suggesting that Hdac2 controls *Acan* levels in PV^+^ cells. By determining Aggrecan expression levels, PV^+^ cells may cell-autonomously modulate PNN dynamics and integrity, thereby regulating the stability of their synaptic inputs [19, 52, 59]. Further, a recent study showed that 4 days of monocular deprivation was sufficient to produce a strong shift in ocular dominance in adult *Acan*^*lox/lox*^ mice injected with AAV-Cre, suggesting that *Acan* expression in the adult brain acts as a brake on neuroplasticity [30]. Therefore, decreasing *Acan* levels in *PV-Cre; Hdac2*^*lox/lox*^ or BRD6688-injected mice likely promotes neuroplasticity, which in turn contributes to stronger retention of extinction memory and reduced spontaneous recovery of fear memory over time. Consistent with this hypothesis, our data show that a brief *Acan* reduction coupled with extinction training was sufficient to decrease the spontaneous recovery of fear memory. Furthermore, we showed that PV^+^ cell-specific *Hdac2* deletion reduced *Acan* transcription selectively in PFC, but not in somatosensory cortex, PV^+^ cells. This surprising regional specificity suggests that PV^+^ cell circuits in different adult cortical areas might differ more, at least in term of epigenetic regulation, than what was previously assumed. In particular, region-specific dynamic epigenetic regulation of PV^+^ cells could increase plasticity of PFC networks compared to primary sensory areas in the adult brain.

In contrast to what we observed in the PFC, the reduced numbers of PV^+^ cells enwrapped by PNN in the BLA of *PV-Cre; Hdac2*^*lox/lox*^ mice was not accompanied by reduced *Acan*/Aggrecan expression, indicating that different molecular organizers might be at play in the two regions. In BLA, PNN enwrap not only PV^+^ cells, but also a population of excitatory neurons [60], therefore PNNs components produced by excitatory neurons may contribute to PNN aggregation around PV^+^ cells. Finally, consistent with previous findings [42], we observed that the expression of the metalloproteases *Adamts8*, *Adamts15* and *Mme* was highly enriched in, but not restricted to, PV^+^ cells. Increased metalloproteases expression might lead to Aggrecan cleavage and PNN disassembly. Whether their expression levels are regulated, directly or indirectly, by *Hdac2* remains to be explored.

Finally, we observed that while *Acan* gene expression in PFC PV^+^ cells was lower in naive *PV_Cre; Hdac2*^*lox/lox*^ and in BRD6688-injected wild-type mice compared to their respective control groups, the difference was lost after extinction training, suggesting that Hdac2 might limit dynamic changes in *Acan* expression. Increased *Acan* expression likely leads to increased PNN aggregation and stabilization around PV^+^ cells [23, 45, 46]. It has been suggested that new PNN formation plays a critical role in memory consolidation, by modulating PV^+^ cell activity; [61] therefore, it is possible that the ability to dynamically regulate *Acan*, and thus PNN formation/stability, and not absolute levels of *Acan*, determines the degree of spontaneous fear memory recovery over time. A recent study showed that Hdac2 S-nitrosylation was upregulated in the hippocampus during the extinction of contextual fear memories. Hdac2 S-nitrosylation caused Hdac2 dislocation from the chromatin and increased chromatin acetylation, thereby priming the expression of neuroplasticity-regulated genes (“transcriptionally permissive state”) [23]. Whether the same epigenetic mechanism is at play in PFC PV^+^ cells during fear extinction learning and whether Aggrecan expression is downstream of such regulation, remains to be investigated. Nevertheless, our findings suggest that Hdac2 levels and/or activity regulate *Acan* expression specifically in PFC PV^+^ cells, but not in somatosensory or BLA PV^*+*^ cells, in adult mice. Therefore, any future study aimed at dissecting the molecular mechanisms linking Hdac2 and *Acan* expression must specifically target adult PFC PV^+^ cells.

Taken together, our data support a model in which the acetylation of PV^+^ cell chromatin primes the *Acan* gene in PFC to be dynamically expressed during extinction learning. Increased Aggrecan expression in turn promotes PNN condensation [23, 45, 46], and modulates PV^+^ cell-mediated inhibition in the PFC contributing to reduced fear expression over time, following extinction training. In addition, acetylation of PV^+^ cell chromatin regulates the ability of PV^+^ cell synapses to remodel their output following extinction training, which could promote the selective inhibition of fear memory traces. Since spontaneous recovery of fear response following extinction training remains an important challenge in exposure therapy, any manipulation that can potentiate the plasticity of PV^+^ cell networks, whether it is through modulating chromatin accessibility or the expression of PNN components, could in turn foster adult brain plasticity and be of future therapeutic interest.

## Material and methods

### Animals

*Hdac2*^*lox/lox*^ (The Jackson laboratory (JAX), *B6.Cg-Hdac2*^*tm1.1Rdp*^*/J*, 022625) mice, in which exons 5 and 6 of *Hdac2* gene, encoding the HDAC-catalytic core of the protein, are flanked by loxP sites, were crossed to the *PV-Cre* (JAX, *B6.129P2-Pvalb*^*tm1(cre)Arbr*^*/J*, 017320) line to generate *PV-Cre*^*+/−*^*;Hdac2*^*lox/lox*^ (*Hdac2* cKO) mice. These lines were maintained through crosses of *PV-Cre*^*+/−*^*;Hdac2*^*lox/+*^ females and *Hdac2*^*lox/lox*^ males to generate *PV-Cre*^*+/−*^*;Hdac2*^*lox/lox*^ and *PV-Cre*^*−/−*^*;Hdac2*^*lox/lox*^ or *PV-Cre*^*−/−*^*;Hdac2*^*lox/+*^ control littermates, or *PV-Cre*^*+/+*^*;Hdac2*^*lox/+*^ females and *Hdac2*^*lox/+*^ males to generate *PV-Cre*^*+/−*^*;Hdac2*^*lox/lox*^ and *PV-Cre*^*+/−*^*;Hdac2*^*+/+*^ control littermates, on a mixed 129sv/C57BL/6J background. Cell specificity of Cre-mediated recombination was analyzed by breeding *PV-Cre* with *RCE*^*EGFP*^ mice (JAX, *Gt(ROSA)26Sor*^*tm1.1(CAG-EGFP)Fsh*^*/Mjax*, 32037). All animals were maintained under a light-dark cycle (12 h light–12 h dark) in a temperature and humidity-controlled room. Food and water were available *ad libitum*. All procedures described here had been approved by the Comité Institutionnel de Bonnes Pratiques Animales en Recherche (CIBPAR) of the Research Center of Sainte-Justine Hospital in accordance with the principles published in the Canadian Council on Animal’s Care’s (Guide to the Care and Use of Experimental Animals).

### Mice genotyping

DNA was extracted from mouse tails and genotyped to detect the presence of Cre alleles and *Hdac2* conditional and wild-type alleles. Polymerase chain reaction (PCR) was performed using either a set of 2 separated primers (F1 5’-TGGTATGTGCATTTGGGAGA-3’ and R1 5’-ATTTCACAGCCCCAGCTAAGA-3’) to identify the *Hdac2* floxed versus the wildtype allele or a set of 3 separate primers (5’-ATTTGGGAGAAGGCCGTTT-3’, 5’-AATTTCACAGCCCCAGCTAAG-3’ and 5′-CGAAATACCTGGGTAGATAAAGC-3′) to assure the absence of the *Hdac2* null allele. Band sizes were of 720 bp for the wild-type, 560 bp for the floxed and 380 bp for the null allele. The 3 separate primers used to detect *Cre* in *PV_Cre* mice were: F1 (5’-CAGCCTCTGTTCCACATACACTCC-3’), F2 (5’- GCTCAGAGCCTCCATTCCCT-3’) and R1 (5’-TCACTCGAGAGTACCAAGCAGGCAGGA GATATC-3’) which generated 400 bp and 526 bp (mutant and wild-type) bands. To detect the presence of the RCE allele, 3 separate primers namely, RCE-Rosa1 (5’-CCCAAAGTCGCTCTGAGTTGTTATC-3’), RCE-Rosa2 (5’GAAGGAGCGGGAGAAATGGATATG-3,) and RCE-Cag3 (5’-CCAGGCGGGC CATTTACCGTAAG-3’) were used, which generated 350 bp and 550 bp bands.

### Immunohistochemistry

Mice of both sexes were anesthetized, then perfused intracardially with PBS followed by 4% (w/v) paraformaldehyde (PFA) in PBS. Intact brains were extracted and post-fixed in 4% PFA/PBS overnight at 4 °C. The tissue was then cryoprotected in 30% (w/v) sucrose (Sigma) in PBS, sectioned coronally at 40 μm on a cryostat (Leica VT100) and stored as floating sections in PBS. For immunohistological analysis, brain sections were blocked in 10% normal goat serum (NGS, Invitrogen, 10000C) in PBS containing 1% (v/v) Triton X-100 for 2 h at room temperature. Primary antibodies were diluted in 5% goat serum in PBS containing 0.1% (v/v) Triton X-100 and incubated 24–48 h at 4 °C. Slices were then washed in PBS (3 ×10’), incubated in the appropriate Alexa-conjugated antibodies in 5% NGS, 0,1% Triton in PBS for 2 h at room temperature, washed again in PBS (3 ×10’), and mounted in Vectashield (Vector Lab, H-1000) before imaging.

The primary antibodies used in this study and their working concentrations are as follows: rabbit monoclonal anti-Hdac2 (1:500; Abcam, 32117, RRID:AB_732777); mouse monoclonal anti-PV (1:2000; Swant, 235, RRID:AB_10000343); rabbit polyclonal anti-PV (1:4000; Swant, PV27, RRID:AB_2631173); guinea pig polyclonal anti-PV (1:1000; Synaptic Systems, 195004, RRID:AB_2156476); mouse monoclonal anti-gephyrin (1:500; Synaptic Systems, 147021, RRID:AB_22325461); rabbit polyclonal anti-aggrecan (1:500; Millipore, AB1031, RRID:AB_90460); chicken polyclonal anti-GFP (1:1000; Abcam, 13970, RRID:AB_300798). To label PNNs, a solution of biotin-conjugated lectin Wisteria floribunda (WFA) (10 µg/ml; Sigma-Aldrich, L1516) was added in the primary antibody solution.

The secondary antibodies used in this study and their working concentrations are as follows: goat anti-chicken Alexa488 conjugated (1:1000; Abcam, ab150169), goat anti-rabbit Alexa633 conjugated (1:400; Life technologies, A21072), goat anti-mouse Alexa555 conjugated (1:1000; Cell Signaling, 4409S), goat anti-mouse Alexa647 conjugated (1:1000; Cell signaling, 4410S), goat anti-rabbit Alexa555 conjugated (1:400; Life technologies, A21430), goat anti-guinea pig Alexa647 conjugated (1:400; Life technologies, AB21450) and Alexa 568-conjugated streptavidin (1:500; Invitrogen, S-11226).

All immunohistological experiments were performed on at least three different sections per brain region per animal. No mice were excluded from the following analysis.

### Confocal imaging

All images were acquired using Leica confocal microscopes (SP8 or SP8-STED). We imaged somatosensory (SSCX) and prefrontal cortex (PFC) layer 5 and basolateral amygdala (BLA) using 20X multi-immersion (NA 0.75) and 63X oil (NA1.4) objectives. We focussed on layer 5 of SSCX and PFC because PV^+^ cells are more abundant in this layer. The 20X objective was used to acquire images to analyze the percentage of: GFP^+^PV^+^/PV^+^ cells (recombination rate), GFP^+^PV^+^/GFP^+^ cells (Cre-recombination specificity), PNN^+^PV^+^/PV^+^ cells and Agg^+^PV^+^/PV^+^ cells. The 63X objective was used to acquire images to analyze the perisomatic innervation (number of perisomatic PV^+^ boutons and gephyrin^+^ punctas). At least three confocal images from three different brain sections were acquired per brain region with z-step size of 2 µm (20X) and 0.5 µm (63X). All the confocal parameters were maintained constant throughout the acquisition of an experiment.

### Image analysis

The number of positive cells (GFP^+^, PV^+^, PNN^+^ and/or Agg^+^) were manually identified and counted using ImageJ-Fiji software. To quantify the number of PV^+^, gephyrin^+^ and PV^+^/gephyrin^+^ punctas, images were exported as TIFF files and analyzed using Neurolucida software. PV^+^ boutons and gephyrin^+^ punctas were independently identified around the perimeter of a targeted cell after selecting the focal plane with the highest soma circumference. At least 4 perisomatic innervated somata were selected in each confocal image. Investigators were blind to the genotypes or conditions during the analysis. Each experiment included both controls and mutant or treated mice.

### Behavioral testing

For all experiments, a camera was mounted above the arena; images were captured and transmitted to a computer running the Smart software (Panlab, Harvard Apparatus) or FreezeFrame software IMAQ 3 (Version 3.0.1.0). The sequence of animals tested was randomized by the genotype. Care was taken to test litters containing both the genotypes specific to the breeding. Room lights were kept low during all procedures.

#### Open field

Each subject (P60) was gently placed at the center of the open-field arena and recorded by a video camera for 10 min. The recorded video file was analyzed with the SMART video tracking system (v3.0, Harvard Apparatus). The open field arena was cleaned with 70% ethanol and wiped with paper towels between each trial. The time spent in the center (45% of the surface) versus the periphery was calculated. Locomotor activity was indexed as the total distance traveled (m).

#### Elevated plus maze

The apparatus consisted of two open arms without walls across from each other and perpendicular to two closed arms with walls joining at a central platform. Each subject (P60) was placed at the junction of the two open and closed arms and allowed to explore the maze during 5 min while video recorded. The recorded video file was analyzed with the SMART video tracking system (v3.0, Harvard Apparatus) to evaluate the percentage of time spent in the open arms (open/(open + closed) × 100) and the number of entries in the open arms as a measure of anxiety-related behavior.

#### Fear conditioning and fear extinction

Fear conditioning and extinction were conducted in an isolated behavior room using standard operant boxes in two different contexts (contexts A and B, respectively). The context A consisted of white walls, a grid floor, and was washed with 70% ethanol before and after each session. The context B consisted of black and white stripped walls, a white plexiglass floor, and was washed with 1% acetic acid before and after each session. Two different fear conditioning protocols [28] were used in this study, as described in details below.

*PV-Cre*^*+/−*^*; Hdac2*^*lox/lox*^ males and their *PV-Cre*^*−/−*^*; Hdac2*^*lox/lox*^ or *PV-Cre*^*−/−*^*; Hdac2*^*lox/+*^ control male littermates, or *Hdac2*^*lox*^ wildtype males, were conditioned using the following protocol. One day prior to fear conditioning (Day 0), mice were allowed to freely explore the context A for habituation. On day 1, mice were conditioned using 5 pairings of the conditioned stimulus (CS duration 5 s, white noise, 80 dB) with a co-terminating unconditioned stimulus (US, 1 s foot-shock, 0.6 mA, inter-trial interval: 30–60 s). On days 2 and 3, conditioned mice were submitted to extinction training in context B. During this training, 12 CS were presented at 30 s intervals on each day. Retrieval of extinction memory and context-dependent fear renewal were tested 7 days later in context B and A, respectively, using 4 presentations of the CS. The retrieval test allowed to quantify the spontaneous recovery of fear memory associated with the cue (CS, conditional stimulus), since it is performed in the same context as extinction learning. The renewal test was performed using the same sound and context of fear acquisition (day 1). The goal of this test was to verify weather extinction of cue-based fear memory was generalized to the context as well, a behavior that is common in very young animals [8, 28, 31].

*PV-Cre*^*+/−*^*; Hdac2*^*lox/lox*^ males and their *PV-Cre*^*+/−*^*; Hdac2*^*+/+*^ control male littermates were conditioned using the following protocol. On day 0, mice were allowed to freely explore the context A for habituation. On day 1, mice were conditioned using 5 pairings of the conditioned stimulus (CS duration 30 s, 50 ms pips repeated at 0.9 Hz, 2 ms rise and fall, pip frequency: 7.5 kHz, 80 dB) with an unconditioned stimulus (US, 1 s foot-shock, 0.6 mA, inter-trial interval: 30–150 s). The onset of the US coincided with the offset of the CS. On days 2 and 3, conditioned mice were exposed to extinction training in context B. During this training, mice were exposed to 12 CS at 30 s intervals on each day. Retrieval of extinction and context-dependent fear renewal were tested 7 days later in context B and A, respectively, using 4 presentations of the CS.

Mice behavior was video-recorded with FreezeFrame software. An experimenter blind to the genotype or experimental conditions scored freezing behavior (defined by complete immobility with the exception of respiratory movements) by measuring the time spent freezing during 30 s following CS presentation. Results presented are the mean of time spent freezing after the presentation of the first 2 CS on the first day of extinction (day 2, CS1-2 = early extinction), the last 2 CS on the second day of extinction (day 3, CS 11-12 = late extinction), the first 2 CS during the retrieval test in the extinction context B (day 10, CS 1-2 = retrieval) and the first 2 CS during the renewal test in the fear acquisition context A (day 10, CS 1-2 = renewal).

Mice were excluded from analysis if they did not show effective fear extinction (freezing more than 80% of time at the end of extinction training, Late extinction = day 3, CS 11-12): Fig. 1b: *n* = 3 control mice excluded, *n* = 0 *PV-Cre*^*+/−*^*; Hdac2*^*lox/lox*^ mouse excluded, Fig. 1g: *n* = 0 vehicle-injected *Hdac2*^*lox*^ mouse excluded, *n* = 1 BRD6688-injected *Hdac2*^*lox*^ mouse excluded, Supplementary Fig. 2: *n* = 0 *PV-Cre*^*+/−*^*; Hdac2*^*+/+*^ mouse excluded, *n* = 1 *PV-Cre*^*+/−*^*; Hdac2*^*lox/lox*^ mouse excluded. Mice were also excluded from analysis if they did not show effective fear acquisition (freezing less than 15% of time at the onset of extinction training, early extinction = day 2, CS 1-2): Supplementary Fig. 2: *n* = 0 *PV-Cre*^*+/−*^*; Hdac2*^*+/+*^ mouse excluded, *n* = 1 *PV-Cre*^*+/*^*; Hdac2*^*lox/lox*^ mouse excluded.

### Viral vector and stereotaxic injections

pAAV.EF1α-DIO-Flag-Hdac2_T2A_GFP (titre – 1E13 GC/mL) was cloned from pCIG-HDAC2-IRES-eGFP [62] and produced as AAV2/9 serotype by the Canadian Neurophotonics Platform. pAAV_EF1α_DIO_eYFP control virus was produced by the same platform (titre – 5E13 GC/mL [injected at 1:6 dilution]). *PV-Cre; Hdac2*^*+/+*^ or *PV-Cre; Hdac2*^*lox/lox*^ mice were used at postnatal day (P) 42-50 for all surgeries. Unilateral viral injections were performed at the following stereotaxic coordinates: +1.9 mm Anterior-Posterior (from Bregma), +0.4 mm Medio-Lateral and 1.6 mm Depth from the cortical surface. Surgical procedures were standardized to minimize the variability of AAV injections. Glass pipette with the virus was lowered at the right depth and kept for 2 min before starting the injections. Each injection volume was kept to 46 nl, and a total of 8 injections per mouse were done for each virus. To ensure minimal leakage into surrounding brain areas and to optimize viral spread in the desired area, injection pipettes were kept in the same position following the injection for 5 min. They were withdrawn at the rate of 0.25 mm/min following the injection. Mice were kept for 3 weeks following viral injections to allow for optimal gene expression. The efficiency of AAV injection was evaluated in each mouse by GFP^+^ cell density. No mouse was excluded in this analysis.

### PFC dissection and dissociation

Mice of both sexes aged P40-P43, were anesthetized with ketamine/xylazine (4:1), transcardically perfused with ice-cold aCSF solution of the following composition (in mM): 62.5 NaCl, 70 sucrose, 2.5 KCl, 25 NaHCO_3_, 1.25 NaH_2_PO_4_, 7 MgCl_2_, 1 CaCl_2_, 20 HEPES and 20 glucose. Brains were then quickly removed, transferred to ice-cold aCSF solution and fixed to a stage allowing to cut 300 μm slices with a VT1200 S, Leica vibratome. Slices were individually transferred to a cutting surface in ice-cold aCSF, where frontal cortex was micro dissected from the slices. Dissected tissue was then dissociated into cell suspension [63] using the Papain dissociation system (Worthington) as per manufacturer’s instructions, with the addition of trehalose as described in [64]. Post trituration, cells were then counted using iNCYTO C-Chip hemocytometers, and resuspended at 150–200 cells/μl in DropSeq Buffer (in mM): x1 HBSS, 7 MgCl_2_, 1 CaCl_2_, 20 glucose, 5% Trehalose and 10% BSA.

### Single cell capture and sequencing

Resuspended cells were run through a custom Drop-Seq setup allowing the capture of single cells and barcoded beads (ChemGenes) within nanoliter droplets as described in the original Drop-seq protocol (http://mccarrolllab.com/dropseq/) [65]. The setup closely resembled the original one [65], with minor modifications. In particular, Medfusion 3500 syringe pumps (Smiths Medical) instead of KD Scientific Legato 100 pumps were used, as well as a different RNase inhibitor (SUPERase• In; catalog no. AM2694; Thermo Fisher Scientific).

Single-cell suspensions (150–200 cells/µl) were run alongside barcoded beads (140 beads µl/1) in 1.7 ml of cell lysis buffer (Tris 200 mM, EDTA 20 mM, 20% Sarkosyl, 20% Ficoll, DTT 50 mM; also containing 50 µl of RNase inhibitor), to produce beads attached to single-cell transcriptomes. Beads attached to single-cell transcriptomes were subsequently washed, reverse-transcribed, treated with exonuclease and counted using a Fuchs-Rosenthal hemocytometer (INCYTO C-Chip Cat-Nr:82030–474), as described in the original Drop-seq protocol [65]. Bead counting was performed using water and bead counting solution (10% polyethylene glycol, 2.5 M NaCl) in a 1:1 ratio as described in Gierahn et al. 2017 [66]. For PCR amplification 4000 beads (approximately 200 single-cell transcriptomes attached to microparticles) were used as input for each PCR. Individual PCR reactions were then pooled to achieve the desired number of single-cell transcriptomes attached to microparticles (STAMPS). In this experiment we used 2000 STAMPS which were cleaned up with AMPure XP (Beckmann Coulter Life Sciences) beads at a ratio of 0.6X prior to sequencing.

Correct size distribution and concentration of complementary DNA was determined using a Bioanalyzer High Sensitivity DNA assay (catalog no. 5067–4626; Agilent Technologies). Library preparation was performed using the Nextera XT DNA Library Preparation Kit (Illumina) with 600 pg input according to the Drop-seq protocol using Illumina i7 primers (N701—N706) together with a custom P5 primer (GCCTGTCCGCGGAAGCAGTGGTATCAACGCAGAGTAC) in the library PCR amplification (see the Drop-seq protocol [65]). Libraries were quality-controlled for size and concentration using the Bioanalyzer High Sensitivity DNA assay. Libraries were quantified using the Universal Library Quantification Kit (KAPA) before sequencing on a NextSeq 550 system (Illumina) at the Institute for Research in Immunology and Cancer (IRIC) in Montreal. Sequencing was performed on a NextSeq 500 system with the read settings R1 = 20 bp, R2 = 63 bp and index = 8 bp.

### Computational analysis of single-cell transcriptomic data

Demultiplexing of raw Illumina sequencing data was performed with bcl2fastq v.2.17 by the Institute for Research in Immunology and Cancer (IRIC) in Montreal. Fastq files were quality controlled (Supplementary Fig. 4) and processed using the Drop-seq computational pipeline, similar to earlier report [67] (Drop-seq alignment cookbook v.1.2, January 2016) using Drop-seq tools v.1.12, to produce digital gene expression (DGE) matrices for each sample [65]. DGE matrices were processed in R (3.5.2) using Seurat (v.2.2.0) [68, 69]. Cells with less than 250 genes expressed and genes detected in less than 3 cells were filtered out from further analysis. Cell cycle scores for filtered cells were calculated using the function CellCycleScoring from Seurat with cell cycle gene lists from Nestorowa et al. [70] (https://satijalab.org/seurat/articles/cell_cycle_vignette.html). Expression values were normalized and scaled to 10,000 transcripts with Seurat’s Normalize function. Normalized expression values were scaled using the ScaleData function from Seurat, regressing out the number of UMIs, Mouse ID, percent mitochondrial genes as well as S and G2M scores from cell cycle prediction.

Cells from eight different mice were then aligned with canonical correlation analysis (CCA) [69]. To do so, we used the RunMultiCCA function on the top 1000 highly variable genes overlapping between the samples. We subsequently ran the AlignSubspace function in Seurat, using the experiment as a grouping variable with an appropriate number of CCA dimensions. These dimensions were derived from the inflection point of the elbow plot generated by Seurat ElbowPlot function, resulting in 15 dimensions. The output from this analysis was then analyzed with RunTSNE. followed by FindClusters function, with a resolution parameter of 1.2. The process resulted in identification of 18 total clusters that were subsequently plotted in 2 tSNE dimensions. Biological cell types in the 18 resulting clusters were annotated manually based on known markers for brain cell types. Violin Plots were plotted using the VlnPlot function from Seurat with normalized gene expression values.

### Cell type identification

Final cell types were attributed based on the identified top markers in each cluster. Clusters containing VGLUT1 were considered to be pyramidal Neurons, while clusters containing GAD1 and GAD2 were considered to be GABAergic interneurons (Fig. 3a, Supplementary Fig. 5). For more in depth analysis of neuron types as well as identification of non-neuronal cell subtypes, we took the top 30 and 50 markers from each cluster and compared them to the DropVis database [71]. The number of markers shared for each cluster was then calculated, and a percentage identity with DropViz clusters calculated.

### Fluorescent multiplex RNAscope

To evaluate *Acan* mRNA expression in naive condition, mice of both sexes were cervically dislocated, brains were dissected, fast-frozen in cold isopentane (~−70 °C) and conserved at −80 °C. To evaluate *Acan* mRNA expression after extinction training, adult males were cervically dislocated 3 h after the end of extinction training on day 4 (late extinction).

Coronal brain sections (20 µm) were cut using a cryostat (Leica Microsystems) and mounted on Superfrost Plus Gold Glass Slides (Fisher Scientific, 22-035-813). Slides were subsequently stored at −80 °C. Probes against *Acan* mRNA (439101), which codes for aggrecan, and *Pvalb* mRNA (421931-C2), which codes for PV, as well as other reagents for ISH and DAPI labeling, were purchased from Advanced Cell Diagnostics (ACD). Tissue pre-treatment, hybridization, amplification and detection were performed according to the *RNAscope Sample Preparation and Pretreatment Guide for Fresh Frozen Tissue using RNAscope Fluorescent Multiplex Assay* manual (ACD). During RNAscope hybridization, positive probes (320881), negative probes (320871), and *acan*/*Pvalb* probes were processed simultaneously. Briefly, the slides were removed from −80 °C and immediately post-fixed in 4% PFA/PB for 15 min before dehydration in 5 min consecutive baths (70% EtOH, 50% EtOH, 2 ×100% EtOH). The slides were air dried and hydrophobic barrier was created around each section with ImmEdge Pen (310018). Protease IV (322336) was added to each section and incubated at room temperature for 30 min followed by two washes in distilled water. For detection, probes were added to each section and incubated for 2 h at 40 °C. Unbound probes were subsequently washed away by rinsing slides in 1X wash buffer (310091) 2 × 2 min. AMP reagents (AMP1 (320852), AMP2 (320853), AMP3 (320854), AMP4A (C1 probes-Alexa-488 and C2 probes-Atto-550) (320855)) were added to each section and incubates for as per the manufacturer’s instructions (*RNAscope Fluorescent Multiplex Reagent Kit PART 2 Manual*), and washed in wash buffer for 2 × 2 min. Sections were stained with DAPI (320858) for 30 s, and then mounted with Prolong Diamond Antifade Mountant (Invitrogen P36961). Images were acquired with a SP8-STED confocal microscope (Leica Microsystems), using a 63X (NA 1.4) objective, voxel size: 0.06 µm × 0.06 µm × 0.299 µm, and deconvolved using Huygen’s express deconvolution option. These experiments were performed using tissue from three different mice for each genotype and/or treatment. To determine the number of *Acan* dots per PV^+^ cell, images were analyzed using a custom-made ImageJ-Fiji macro. Briefly, a dark region was selected on each focal plane to measure the mean gray value (MGV) as background (BCKG). Then, for each channel and each focal plane, the BCKG value was subtracted, and intensity value of each pixel was readjusted by multiplying it with 65535/(65535-BCKG). Then, each PV^+^ cell was manually selected based on the presence of *Pvalb* puncta, filtered with the Gaussian Blur 3D option and analyzed using the 3D objects counter (threshold = 4500, minimal voxel size = 6). Results are presented as mean of the number of objects per PV^+^ cell ±s.e.m. No mice were excluded from these analysis.

### Drug treatment

BRD6688 (Glixx Lab, #GLXC-05908) was dissolved in DMSO (1% of the total resultant solution) and then diluted in 30% Cremophor/69% in physiological saline (H_2_O containing 0.9% NaCl (Hospira, 04888)), for a final dosage solution of 1 mg/kg. Vehicle solutions consisted of the aforementioned solution without the compound. Solutions were prepared immediately before injection and administered by an investigator blind to the genotype to either *Hdac2*^*lox*^ wildtype or *PV-Cre*^*+/−*^*; Hdac2*^*lox/lox*^ via intraperitoneal injection 6 h prior to extinction training performed by a second investigator blind for both the genotype and treatment. Mice were randomly allocated to vehicle or BRD6688 injection.

### siRNA-delivering peptides

TARBP-BTP RNA-binding protein was produced by the National Research Council Canada (NRC), Montreal, Quebec, Canada, as described previously [44]. The original pET28a-His-pTARBP-BTP coding plasmid was provided by CSIR-CCMB. TARBP-BTP:siRNA complex were prepared as previously described [44, 45]. Silencer Select *Acan* mouse targeting siRNA (Life technologies, #s61116) and Silencer Select Negative Control siRNA (Life technologies, #4390843) were resuspended at 200 µm with nuclease-free water. For in vivo delivery, TARBP-BTP and siRNA were drop wise mixed together at 5:1 mole ratio. The complex was incubated 20 min at room temperature and administered intravenously (i.v.) via the lateral tail vein at a dose of 20 nmol:4 nmol TARBP-BTP:siRNA in a total volume of 150 µL per mouse. 48 h after injection, 3 P60 *Hdac2*^*lox*^ mice injected with siCtrlNeg and 4 P60 *Hdac2*^*lox*^ mice injected with siAcan were cervically dislocated, brains were dissected and fast-frozen in cold isopentane (~−70 °C) and conserved at −80 °C before proceeding with Fluorescent multiplex RNAscope. To investigate *Acan* knockdown effect on spontaneous recovery of fear memory after extinction training, 21 *Hdac2*^*lox*^ were fear-trained by a second investigator blind for the treatment using the following protocol. On day 0, mice were allowed to freely explore the context A for habituation. On day 1, mice were conditioned using 5 pairings of the conditioned stimulus (CS, duration 5 s, white noise, 80 dB) with a co-terminating unconditioned stimulus (US, 1 s foot-shock, 0.6 mA, inter-trial interval: 30–60 s). On day 2, 11 mice were i.v. injected with TARBP-BTP:siCtrlNeg and 10 mice were i.v. injected with TARBP-BTP:siAcan at a dose of 20 nmol:4 nmol per mouse. On days 3 and 4, conditioned mice were subjected to extinction training in context B. During this training, mice were presented with 12 CS at 30 s intervals on each day. Spontaneous recovery of fear memory (retrieval of extinction memory test) and context-dependent fear renewal (renewal test) were tested 7 days later in context B and A, respectively, using 4 presentations of the CS. Data analysis was performed by an investigator blind to the treatment and as described above. Mice were randomly allocated to TARBP-BTP:siCtrlNeg or TARBP-BTP:siAcan injection.

### Statistical analysis

No statistical methods were used to predetermine sample size. Each experiment included wild type and mutant littermate mice, or mice injected with vehicle/ TARBP-BTP:siCtrlNeg and BRD6688/ or TARBP-BTP:siAcan. Experiments were performed using multiple litters. Statistical analysis was performed with Prism 6.01 (GraphPad software). Data were systematically tested for normal distribution, with the D’Agostino & Pearson omnibus normality test. Differences between two groups of normally distributed data with homogenous variances were analyzed using parametric student’s t-test, while not normally distributed data were analyzed with the Mann–Whitney test. To evaluate genotype effects on PV^+^Gephyrin^+^ perisomatic puncta in naive mice and in mice following extinction training, two-ways ANOVA test was performed followed by Sidak *posthoc* test (variables: genotype, treatment). To evaluate the effect of virally-mediated re-expression of *Hdac2* on Aggrecan and PNN, Kruskall–Wallis test was performed followed by Dunn’s multiple comparison test. For fear conditioning analysis, we evaluated the extinction rate by Repeated measures two-ways ANOVA, while freezing time at extinction retrieval and fear renewal tests were analyzed with unpaired two-tailed t-test, if normally distributed and Mann–Whitney if not. Results were considered significant for values of *P* < 0.05. Data are presented as mean ± standard error of mean.

### Supplementary information

Supplemental Material

## Data Availability

All data are available upon request.
